# Metabolomic Study of Flavonoids in *Camellia drupifera* under Aluminum Stress by UPLC-MS/MS

**DOI:** 10.3390/plants12071432

**Published:** 2023-03-24

**Authors:** Yi Wang, Junsen Cheng, Shanglin Wei, Wei Jiang, Yongquan Li, Wei Guo, Wenkui Dai, Boyong Liao

**Affiliations:** College of Horticulture and Landscape Architecture, Zhongkai University of Agriculture and Engineering, Guangzhou 510225, China

**Keywords:** *Camellia drupifera*, flavonoid metabolites, aluminum stress, UPLC-MS/MS

## Abstract

Aluminum (Al) affects the yield of forest trees in acidic soils. The oil tea plant (*Camellia drupifera* Lour.) has high Al tolerance, with abundant phenolic compounds in its leaves, especially flavonoid compounds. The role of these flavonoids in the Al resistance of oil tea plants is unclear. In this metabolomic study of *C. drupifera* under Al stress, ultra-pressure liquid chromatography coupled with tandem mass spectrometry (UPLC-MS/MS) was utilized to identify metabolites, while principal component analysis, cluster analysis, and orthogonal partial least squares discriminant analysis were applied to analyze the data on the flavonoid metabolites. The leaf morphology of *C. drupifera* revealed significant damage by excess aluminum ions under each treatment compared with the control group. Under Al stress at 2 mmol/L (GZ2) and 4 mmol/L (GZ4), the total flavonoid content in *C. drupifera* leaves reached 24.37 and 35.64 mg/g, respectively, which are significantly higher than the levels measured in the control group (CK) (*p* < 0.01). In addition, we identified 25 upregulated and 5 downregulated metabolites in the GZ2 vs. CK comparison and 31 upregulated and 7 downregulated flavonoid metabolites in GZ4 vs. CK. The results demonstrate that different levels of Al stress had a significant influence on the metabolite profile of *C. drupifera*. It was found that the abundance of the 24 differential flavonoid metabolites was gradually elevated with increasing concentrations of Al stress, including catechin, epicatechin, naringenin-7-glucoside, astilbin, taxifolin, miquelianin, quercitrin, and quercimeritrin. Moreover, the most significant increase in antioxidant activity (about 30%) was observed in *C. drupifera* precultured in leaf extracts containing 7.5 and 15 μg/mL of active flavonoids. The qRT-PCR results showed that the expression levels of key genes involved in the synthesis of flavonoids were consistent with the accumulation trends of flavonoids under different concentrations of Al. Therefore, our results demonstrate the key role of flavonoid compounds in the oil tea plant *C. drupifera* in response to Al stress, which suggests that flavonoid metabolites in *C. drupifera*, as well as other aluminum-tolerant plants, may help with detoxifying aluminum.

## 1. Introduction

Oil tea is the collective name for plants in *Camellia* with high oil content in the seed kernels [1,2,3]. The species of oil tea gradually evolved into categories such as *C. oleifera* and *C. drupifera* Lour. under the action of environment and inherent genomics [1,2,3]. *C. drupifera* Lour. is cultivated in South China specifically because of its tall tree shape, long growth period, large fruits, and high oil yield, as well as high ornamental value, which is based on its luxuriant white flowers when in bloom [1]. *C. drupifera* is genetically close to *C. oleifera* [2,3], but has a higher fruit yield and oil content than both *C. oleifera* and *C. semiserrata*, and its planting area ranks among the top three in China [4], thus making it a woody oil plant with important economic value in South China. However, South China is a region with a high incidence of acid rain. Normally, aluminum (Al) exists in the form of insoluble silicates and aluminum oxides under neutral or moderately acidic soil conditions, but when the soil is eroded by acid rain or other factors that cause its pH to drop below 5.5, some of the insoluble aluminum will be leached and dissolved as Al^3+^, Al(OH)^2+^, and Al(OH)^2+^ [5,6]. When in a free state, aluminum ions can be absorbed by plant roots, resulting in plants showing symptoms of aluminum toxicity [5,6], and their growth and normal metabolism are affected [7,8].

Because of the increasing extent of acid rain in the southern region, along with the misuse of acidic fertilizers and the existence of irrational farming practices, aluminum in the acidic red soil areas of southern China is subject to leaching [9,10], generating aluminum ions in the free state. These are toxic, inhibiting vegetative growth and plant tissue development of *C. drupifera*, and the growth and yield of *C. drupifera* are limited by the toxic effects of excess aluminum ions. There have been many reports on the inhibition of growth and development of oil tea species by acidic aluminum stress. For example, Huang et al. [11], using the oil tea plant ‘Huajin’ (*C. oleifera*), found that the number of lateral roots and the growth and development of root systems of seedlings were significantly inhibited by 4 mmol/L aluminum stress treatment for 90 days. In addition, aluminum uptake and tolerance mechanisms were investigated, and the net photosynthetic rate, stomatal conductance, and transpiration rate of seedlings were found to decrease by 14.8%, 17.1%, and 13.9%, respectively, under this treatment [11]. In addition, Zhou et al. [12] characterized the effect of phosphorus-aluminum coupling on the growth and physiological indexes of ‘Huajin’ oil tea seedlings, and Qu et al. [13] explored the physiological mechanism of phosphorus in *C. oleifera* seedlings for the purpose of alleviating aluminum toxicity. Although several advances have been achieved in research into the physiological mechanisms of oil tea in response to Al stress, research has mainly focused on *C. oleifera*, and the molecular mechanisms of *C. drupifera* in response to Al stress remain unclear.

In the long process of evolution, a large number of metabolites with different biological functions have been synthesized by plants in response to stimulation by a changeable environment [14]. Plants have a wide range of habitats and are usually exposed to a variety of abiotic stresses; thus, they differ in their morphology and metabolism, resulting in different adaptations to and ecotypes within an environment [15,16]. Plants contain primary and secondary metabolites, and secondary metabolites are involved in the environmental response of resistance to abiotic and biotic stress. Some substances can improve the stress resistance of plants [16]. Flavonoids are an important family of secondary metabolites involved in plant development and defense, with a variety of functions such as antioxidant and antibacterial activity as well as free radical scavenging, and they are also believed to enhance tolerance to abiotic stresses [17,18]. For example, flavonoids are involved in ultraviolet (UV-B) stress [18,19] and drought resistance [20,21], and may play a functional role in heat acclimation and cold tolerance in plants [22,23]. In recent years, there has also been growing interest in the functional role of flavonoids in metal stress [24,25]. Metal stress upregulates the expression of the structural genes *PAL* (phenylalanine ammonia-lyase), *CHS* (chalcone synthase), *DFR* (dihydroflavonol reductase), and *F3H* (flavanone 3-hydroxylase), as well as the transcription factor MYBA1, thereby increasing the accumulation of flavonoid compounds while excessive metal ions suppress their accumulation, as determined by HPLC combined with qRT-PCR [26,27]. The stimulation of CHS activity has been demonstrated in response to copper and cadmium [28,29], while an increase in PAL activity has been revealed in plants exposed to cadmium and lead [30]. Furthermore, liquid chromatography electrospray ionization-mass spectrometry (LC-ESI-MS/MS) was used to analyze the flavonoid composition of two lupine species, *Lupinus albus* and *L. angustifolius*, after lead stress, and Pb (at 150 mg/L) was found to cause significant changes in the levels of 21 flavonoid conjugates [31]. Of these, two compounds, 2′-hydroxygenistein glucoside and malonylated 2′-hydroxygenistein 7-O-glucoside, were found to increase by 219- and 85-fold, respectively, under the 150 mg/L Pb stress treatment [31].

As a common abiotic stress factor in soil, Al stress, similarly to cadmium, lead, and copper stresses, seriously affects plant growth, development, and metabolism [11,32]. Flavonoids have been demonstrated to have Al binding affinity and to carry out detoxification during Al resistance by forming Al chelates or by scavenging reactive oxygen species (ROS) [21,27,33]. *Eucalyptus* is a highly Al-resistant tree because of its secretion of the flavonoid compound oenothein B from the roots of *E. camaldulensis*, and the exogenous application of oenothein B promotes root growth in *Arabidopsis* under Al stress [34]. Moreover, proanthocyanidins are important flavonoid compounds that can prevent the binding of Al ions to root cells by forming metal complexes, while the application of flavan-3-ol from tea can greatly reduce the accumulation of Al ions in soybean root tips [35]. Su et al. [27] found that MsMYB741 transcriptionally activates the expression of MsPAL1 and MsCHI, increases the accumulation of flavonoids in the roots and secretions from root tips, and enhances the ability of alfalfa to scavenge reactive oxygen species (ROS) and H_2_O_2_, thus increasing the resistance of alfalfa to aluminum. To address the scientific question of related mechanisms, it is necessary to elucidate the possible functions of Al-induced flavonoids in *C. drupifera*. In the current study, we explored the question of the role of flavonoids in the amelioration of Al toxicity in plants. The aforementioned findings have demonstrated the important role of flavonoids in plant resistance to Al, so this study was conducted to explore the metabolism of flavonoids in *C. drupifera* in response to Al stress by UPLC-MS/MS, with refinement of the metabolic biological pathways of flavonoids in oil tea plants under Al stress.

## 2. Results

### 2.1. Morphological Characteristics and Total Flavonoid Content under Different Concentrations of Aluminum in C. drupifera

**Figure 1:**
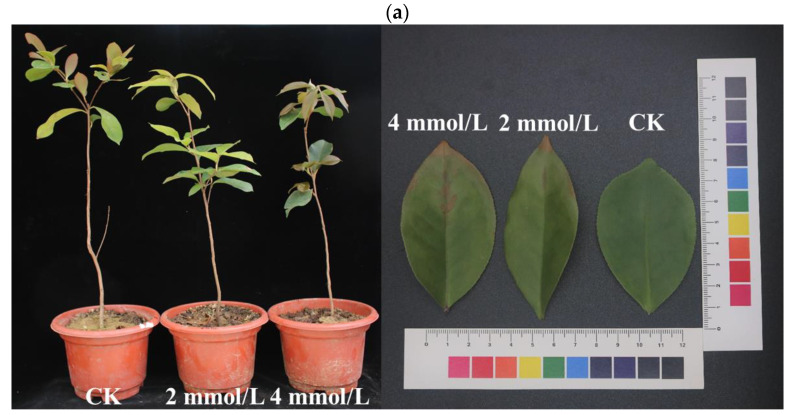

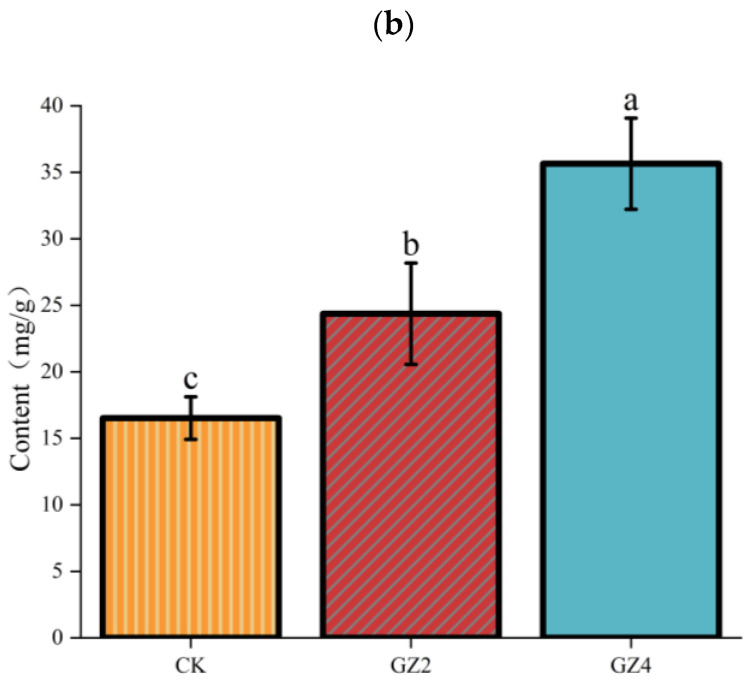
Responses of *C. drupifera* to aluminum treatment. (**a**) Morphological characteristics and (**b**) total flavonoid content under different concentrations of aluminum. In the histogram, different letters indicate statistical significance (*p* < 0.05).

After four weeks of the aluminum stress treatment, the growth of *C. drupifera* seedlings in the treatment group was significantly inhibited, and *C. drupifera* leaves exhibited significant damage by excess aluminum ions in each treatment compared with the control group (Figure 1a). As shown in Figure 1b, the total flavonoid content was significantly higher in *C. drupifera* leaves from the 4 mmol/L Al stress (GZ4) and 2 mmol/L Al stress (GZ2) treatment groups than in the non-stressed control leaves, reaching a level of 35.64 and 24.37 mg/g (fresh weight), respectively. The high accumulation of total flavonoids confirmed that Al stress promotes the synthesis of flavonoid compounds in *C. drupifera* seedlings.

### 2.2. Comprehensive Analysis of Flavonoid Compounds

**Figure 2:**
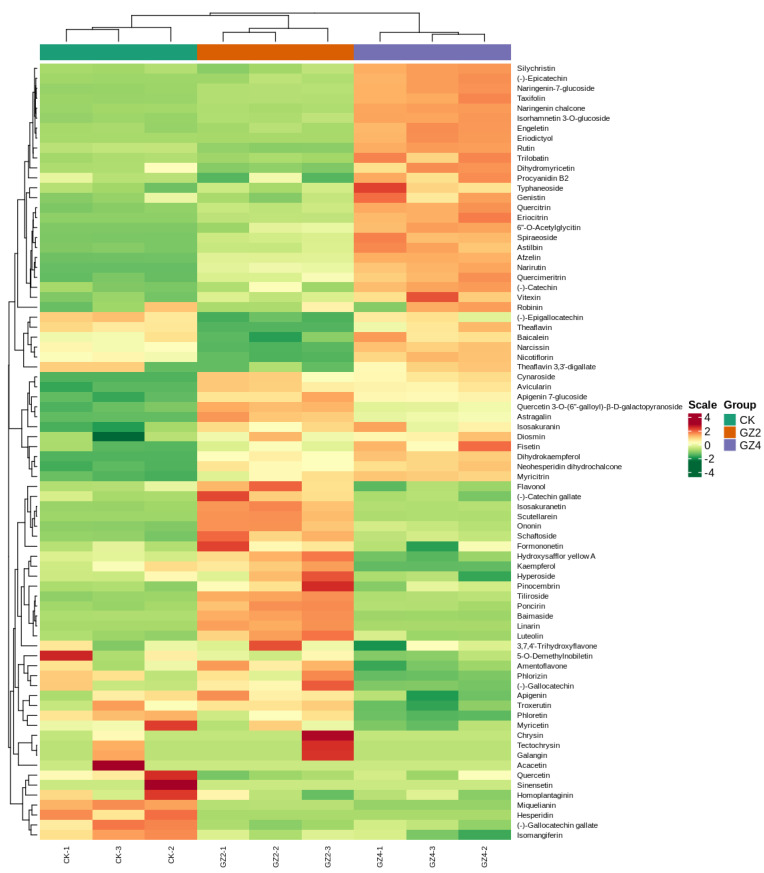
Heatmap of 78 flavonoid metabolites. The data on the flavonoid metabolite contents were normalized for hierarchical cluster analysis. Each column represents one sample, while each metabolite is represented by one row. Red color in the scale bars indicates an increase in relative metabolite abundance, while green color in the scale bars indicates a decrease, with the magnitude of change according to the scale (log_2_ (fold change)).

Widely targeted metabolite analysis based on UPLC-MS/MS was performed to comprehensively profile flavonoids in the leaves of *C. drupifera* after Al stress. According to the Metware Metabolite Database (MWDB) (MetWare, Wuhan, China), which was constructed using standards, the flavonoid metabolites of *C. drupifera* were determined qualitatively and quantitatively through triple quadrupole screening of ions and the signal intensity characteristics of the detected ions. In total, 78 flavonoid metabolites, including 22 flavonols, 17 flavones, 9 flavanones, 6 flavanols, 6 flavanonols, and 5 chalcones, were detected in the leaves of *C. drupifera* under aluminum stress (Figure 2, Table S1).

### 2.3. Principal Component Analysis (PCA) of C. drupifera Samples

**Figure 3:**
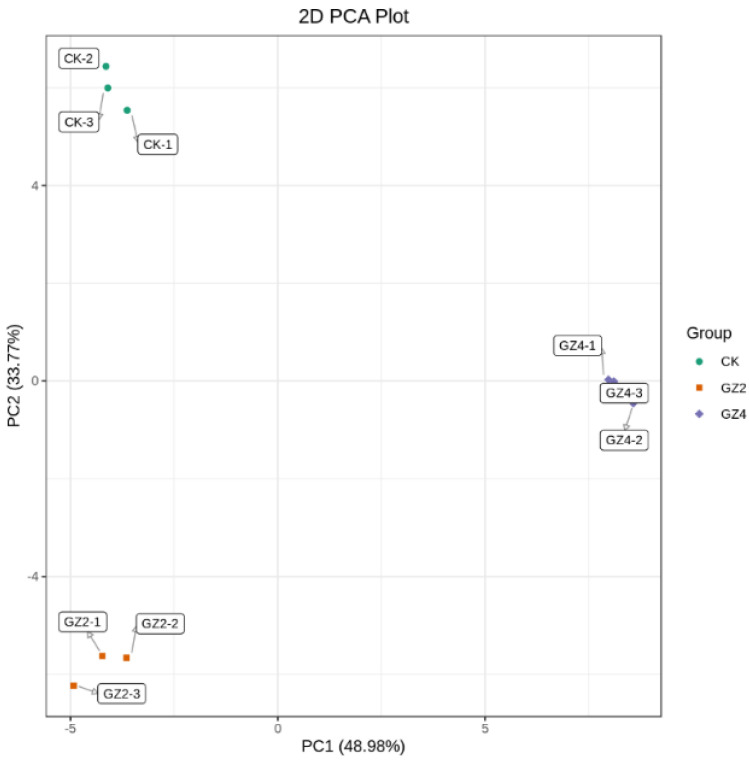
PCA analysis of the flavonoid metabolites in the leaves of *C. drupifera* under aluminum stress.

In our experiments, PCA analysis of *C. drupifera* samples was performed to illustrate the overall metabolic differences between groups and the magnitude of the variation between samples within a group. The PCA results demonstrate a significant trend of separation of the metabolomes between the groups, indicating significant differences in metabolomes among the *C. drupifera* treatment groups. The principal component scores indicate that PC1 and PC2 explain 48.98% and 33.77%, respectively, of the variability in the samples, with a total contribution rate of 82.75% (Figure 3). The three groups of *C. drupifera* samples treated with different concentrations of aluminum were clearly separated and highly reproducible, with close clustering of the biological replicates, which indicates the high reproducibility and scientific validity of the data.

### 2.4. Analysis of Differential Flavonoid Metabolites by OPLS-DA

**Figure 4:**
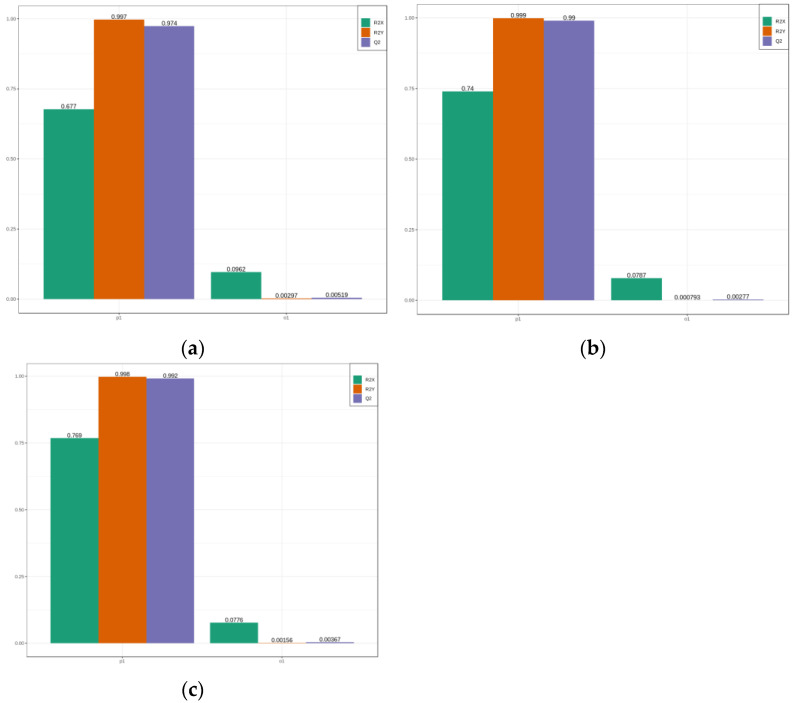
OPLS-DA model plots for (**a**) GZ2 vs. CK; (**b**) GZ4 vs. CK; (**c**) GZ4 vs. GZ2.

The advantage of the OPLS-DA model over PCA analysis is that it allows for maximum differentiation between the groups, thus facilitating the detection of differential metabolites [17]. Q^2^ represents the predictive power of the model, and Q^2^ > 0.9 is usually considered to indicate an excellent model [36]. The metabolomic data of the 78 flavonoids in this research were examined according to the OPLS-DA model, and significant differences were detected among the *C. drupifera* samples. In the comparison between the 2 mmol/L aluminum stress treatment (GZ2) and the control group (CK), R^2^ X = 0.667, R^2^Y = 0.997, and Q^2^ = 0.974 (Figure 4a). In the comparison between the 4 mmol/L aluminum stress treatment (GZ4) and the control group, R^2^ X = 0.74, R^2^Y = 0.999, and Q^2^ = 0.99 (Figure 4b); and in the comparison between GZ4 and GZ2, R^2^ X = 0.677, R^2^Y = 0.997, and Q^2^ = 0.974 (Figure 4c). The Q^2^ values of all the comparisons were close to 1, indicating that the models have high stability and reliability. These samples were clearly separated in the OPLS-DA model score plot, underlining the significant divergence between the flavonoid metabolic profiles of *C. drupifera* samples treated with different concentrations of aluminum.

### 2.5. Screening and Classification of Differential Flavonoid Metabolites

**Figure 5:**
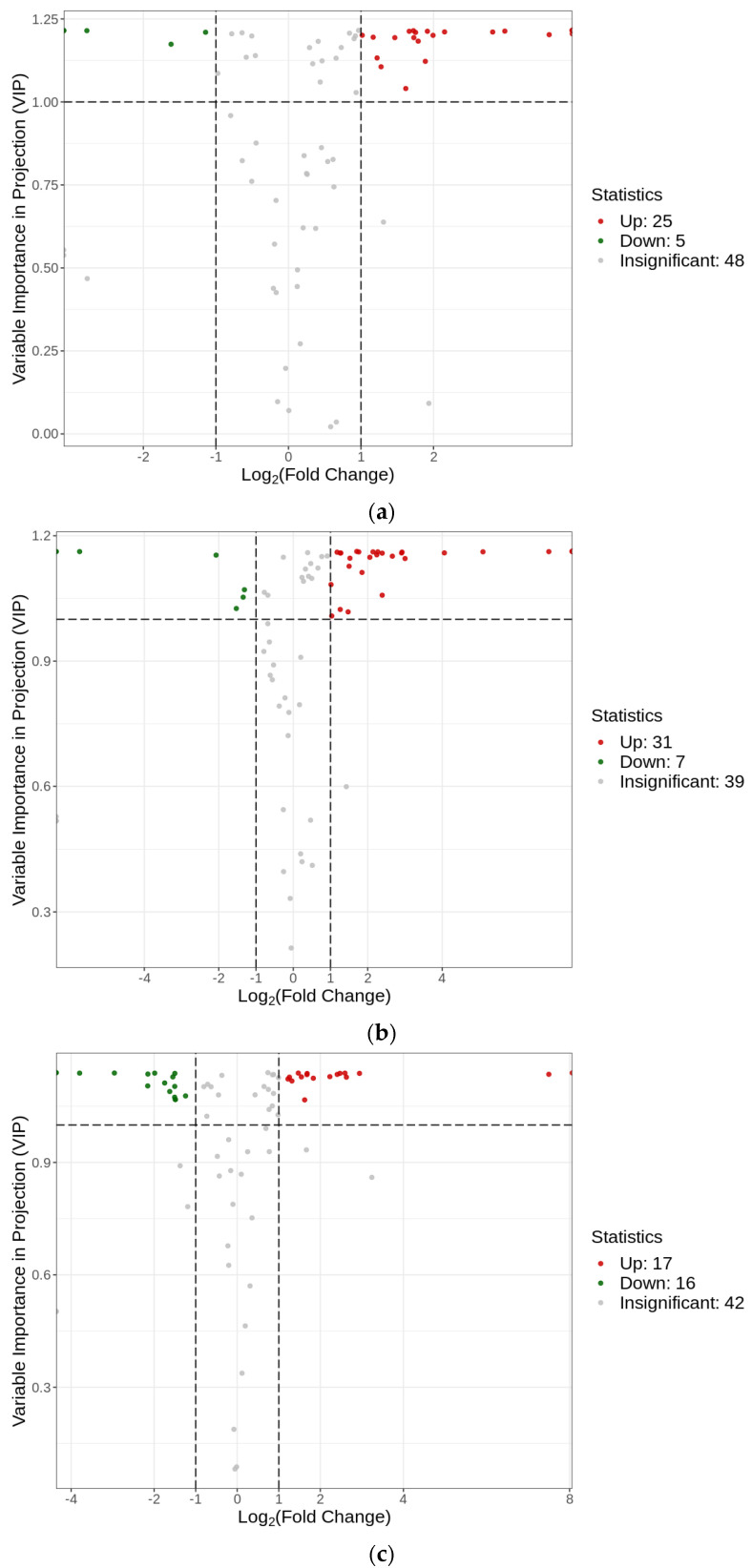
Volcano maps of differential metabolites. (**a**) GZ2 vs. CK; (**b**) GZ4 vs. CK; (**c**) GZ4 vs. GZ2.

**Figure 6:**
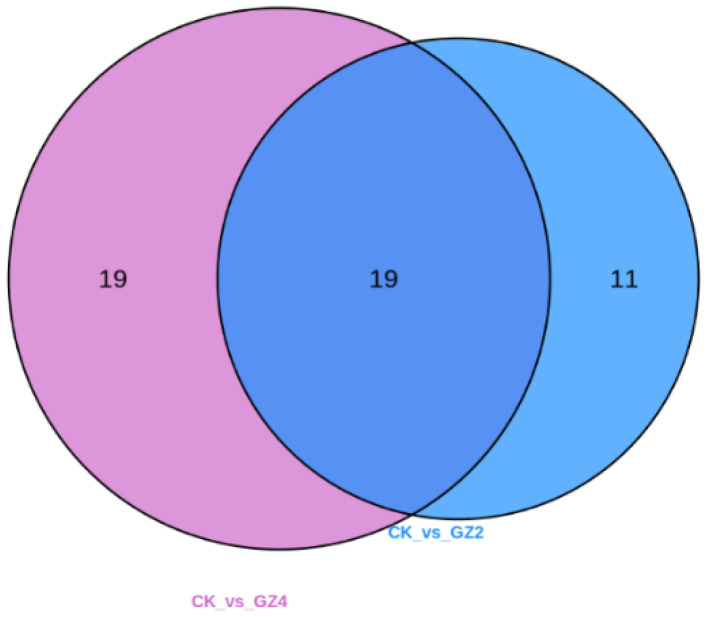
Venn diagram showing the numbers of metabolites in GZ2 vs. CK and GZ4 vs. CK.

To accurately screen for differential flavonoid metabolites between the aluminum stress treatment and control groups, a combination of fold change and variable importance in projection (VIP) values in the OPLS-DA model was utilized in our study to screen for differential flavonoid metabolites, according to the method described by Wang et al. [17]. Differential flavonoid metabolites were selected according to the following criteria: significant fold change of ≥2 or ≤0.5, and VIP ≥ 1. Figure 5 displays the screening process for the differential metabolites. The results of this process of screening differential flavonoid metabolites from the aluminum stress treatment groups and the control group are illustrated as a Venn diagram (Figure 6), and the differential metabolites are listed in Table 1. Through comparisons, 30 significantly different flavonoid metabolites between GZ2 and CK (25 upregulated and 5 downregulated) and 38 significantly different flavonoid metabolites between GZ4 and CK (31 upregulated and 7 downregulated) were identified (Table S2). These results indicate that 19 of these flavonoid metabolites were found in the comparisons between GZ2 and CK and between GZ4 and CK (Figure 6 and Table 1).

### 2.6. Trends of Differential Flavonoid Metabolites under Aluminum Stress Treatments at Different Concentrations

**Figure 7:**
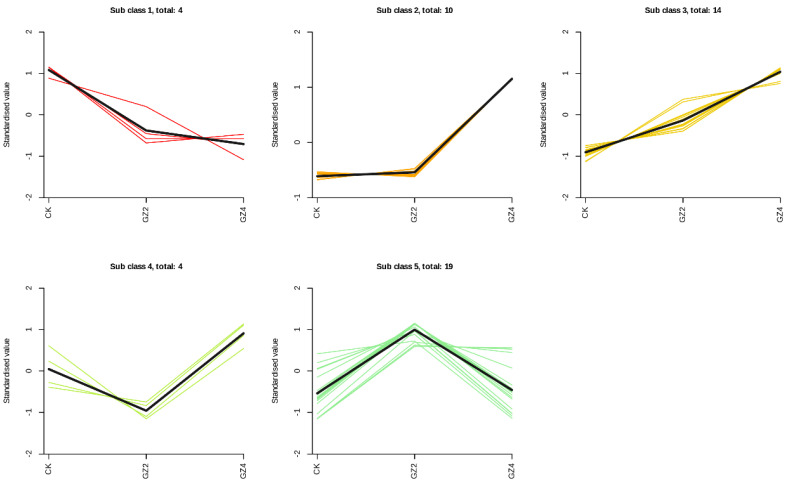
K-means cluster analysis showing the dynamic accumulation of differential metabolites under different concentrations of aluminum.

K-means clustering was used to further classify all the differential metabolites into five subclusters (Figure 7). Subcluster 1 contained four metabolites, which gradually decreased in *C. drupifera* leaves with increasing concentrations of aluminum. Subclusters 2 and 3 contained 24 metabolites, whose contents constantly increased in *C. drupifera* leaves with increasing concentrations of aluminum and reached the highest levels at a concentration of 4 mmol/L aluminum. This indicates that the flavonoid metabolites in subclusters 2 and 3 are closely related to aluminum resistance and are key compounds for alleviating aluminum ion toxicity in *C. drupifera*. The representative compounds in this group include the flavonoids naringenin-7-glucoside, astilbin, taxifolin, and quercimeritrin. Subcluster 4 consisted of 4 metabolites, the levels of which decreased under 2 mmol/L aluminum treatment and then increased greatly under 4 mmol/L aluminum stress treatment. Interestingly, subcluster 5 contained 19 metabolites whose levels greatly increased under 2 mmol/L aluminum stress treatment and then decreased under 4 mmol/L aluminum stress treatment, suggesting that these metabolites may contribute to the regulation of aluminum stress in *C. drupifera* under low concentrations.

### 2.7. Enrichment Analysis of Differential Flavonoid Metabolites

**Figure 8:**
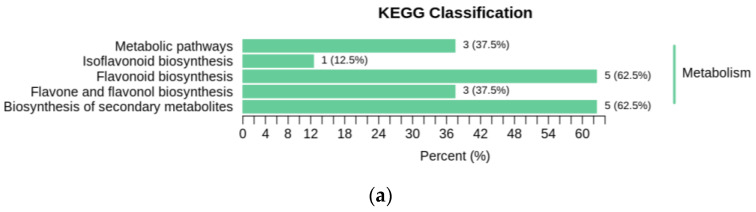

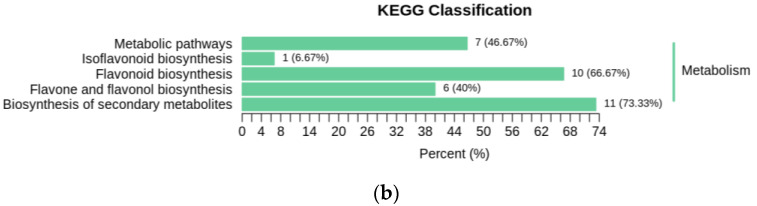
KEGG enrichment analysis of the differential metabolites in the comparisons of (**a**) GZ2 vs. CK and (**b**) GZ4 vs. CK.

Different flavonoid metabolites can play unique roles in organisms, forming distinct metabolic pathways and biological pathways. A total of 30 flavonoid metabolites that were significantly different between GZ2 and CK (25 upregulated, 5 downregulated) and 38 flavonoid metabolites that were significantly different between GZ4 and CK (31 upregulated, 7 downregulated) in *C. drupifera* under aluminum stress were annotated using the Kyoto Encyclopedia of Genes and Genomes (KEGG) database. The aforementioned annotated metabolites in each comparison group are shown in Figure 8a,b. The KEGG classification results and enrichment analysis revealed that the significantly differential flavonoid metabolites were distributed in pathways that included flavonoid biosynthesis (ko00941), the biosynthesis of secondary metabolites (ko01110), metabolic pathways (ko01100), biosynthesis of flavones and flavonols (ko00944), and isoflavonoid biosynthesis (ko00943).

### 2.8. Effect of Flavonoid Pretreatment on Antioxidant Activity

**Figure 9:**
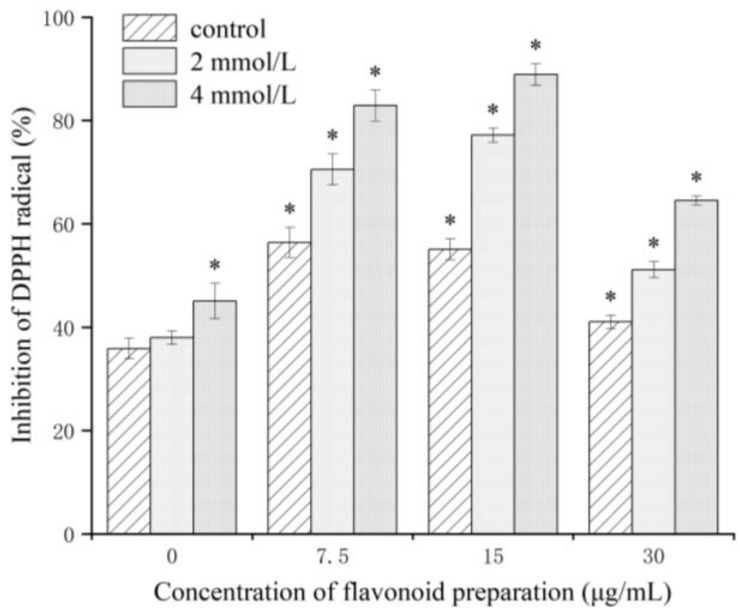
Effect of flavonoid pretreatment on antioxidant activity, expressed as the percentage of inhibition of DPPH radicals in *C. drupifera* leaves. * indicates statistical significance (*p* < 0.05).

To test whether the effect of flavonoid preincubation on Al stress tolerance in *C. drupifera* seedlings was related to the potential antioxidant properties of flavonoids, we calculated the capacity of extracts from *C. drupifera* leaves to scavenge stable DPPH free radicals [26]. Leaf extracts were found to be effective in scavenging DPPH radicals in *C. drupifera* plants treated with 4 mmol/L AlCl_3_-6H_2_O, which were preincubated with flavonoid extracts. As seen in Figure 9, the antioxidant activity is positively correlated with the concentration of the flavonoid preparation. The most significant increase in antioxidant activity (about 30%) was observed in *C. drupifera* precultured in leaf extracts containing 7.5 and 15 μg/mL of active flavonoids. After preincubation and flavonoid preparation, the antioxidant activity was consistently higher in the stressed leaf samples than in the non-stressed controls.

### 2.9. Validation of Metabolomic Data by qRT-PCR Expression Analysis of Key Flavonoid Synthesis Genes

**Figure 10:**
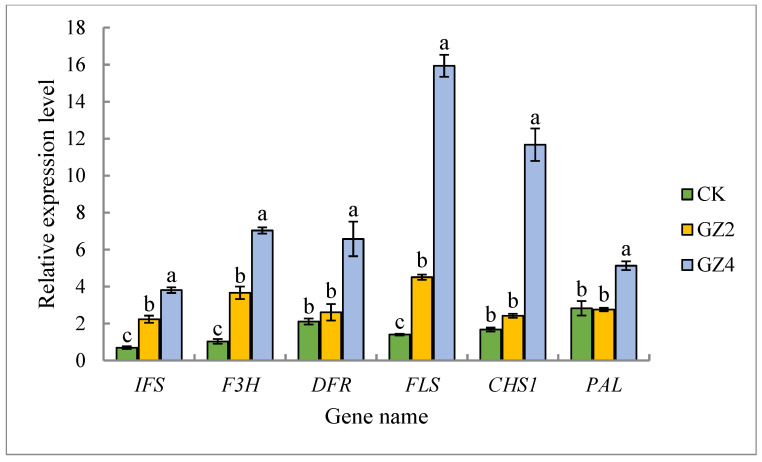
Results of qRT-PCR analysis of the six genes (*IFS*, *F3H*, *DFR*, *FLS*, *CHS1*, and *PAL*) in the flavonoid biosynthetic pathway of *C. drupifera* under different concentrations of Al. Different letters indicate statistical significance (*p* < 0.05).

Analysis by qRT-PCR was used to compare the differences in the relative expression of six genes (*IFS*, *F3H*, *DFR*, *FLS*, *CHS1*, and *PAL*) involved in the synthesis of flavonoids of *C. drupifera* (Figure 10). The qRT-PCR results showed that the expression levels of the genes that encode key enzymes involved in the synthesis of flavonoids were consistent with the trends of the relative flavonoids contents in *C. drupifera* under different concentrations of Al. These results indicate that the measured metabolomic data are valid and reliable.

## 3. Discussion

The environment is one of the most important factors affecting the biosynthesis of secondary metabolites in plants. Elemental Al is a major component of mineral soils, and is present in various primary minerals [10,37]. Aluminum becomes soluble in soils at pH < 5.5, and soluble aluminum in the form of Al^3+^ is readily taken up by plant roots, even when it is only present in micromolar concentrations. Plant growth is thus inhibited, and subsequent nutrient and water uptake is reduced, leading to decreases in fruit yield [38,39,40]. Flavonoids, as an important class of secondary metabolites in plants, exercise important functions in the process of plant-environment interactions. They form part of the plant defense system, playing an important role when plants are subjected to single or multiple stresses such as metal ions, salt, temperature, and drought [21,41,42]. Our current data show that flavonoids also play an important role in the resistance of oil tea to aluminum stress, and 78 flavonoids were screened from the leaves of *C. drupifera* subjected to aluminum stress. The results of the metabolomic study of samples treated with different concentrations of aluminum, alongside those of PCA and OPLS-DA, demonstrate that the three samples are clearly differentiated. When we selected the significantly differential flavonoid metabolites using the criteria of a fold change of ≥2 or ≤0.5 and VIP ≥ 1, 49 flavonoid compounds were found to exhibit significant differences in the level of accumulation under the 2 and 4 mmol/L Al stress treatments relative to the control group, in which 40 flavonoid metabolites were significantly upregulated and nine were downregulated. These experiments demonstrated that growth under the 2 and 4 mmol/L aluminum stress treatments resulted in *C. drupifera* with a significantly higher flavonoid content. This result is similar to our determination of the total flavonoid content. The qRT-PCR results showed that the expression levels of the key genes involved in the synthesis of flavonoids were consistent with the accumulation trends of flavonoids under 2 and 4 mmol/L Al stress treatments. Therefore, more than 80% of the differential flavonoids were significantly upregulated (40 out of 49), and 24 differential flavonoid metabolites were gradually upregulated with increasing concentrations of Al. This demonstrates the key role of flavonoid compounds in the woody oil plant *C. drupifera* in response to Al stress, thus suggesting that flavonoid metabolites in *C. drupifera*, as well as in other aluminum-tolerant plants, may help in detoxifying aluminum.

Flavonoids have been reported to play an important role in plant resistance to aluminum by forming aluminum-chelating complexes or by scavenging free radicals through their Al-binding affinity [27,33,43]. Proanthocyanidins are important flavonoid compounds that can prevent the binding of Al ions to root cells by forming metal complexes, while the application of flavan-3-ol from tea greatly reduces the accumulation of Al ions in soybean root tips [35]. In the present study, we identified 25 upregulated and 5 downregulated flavonoid metabolites between GZ2 and CK and 31 upregulated and 7 downregulated flavonoid metabolites between GZ4 and CK. The biosynthesis of flavonoids was more significantly enriched in the comparison of GZ4 vs. CK than that of GZ2 vs. CK. Many of the highly accumulated flavonoid compounds identified in the GZ2 vs. CK and GZ4 vs. CK comparisons have not previously been reported in the literature as functioning in resistance to Al stress, including 11 flavanones (eriocitrin, hesperidin, pinocembrin, narirutin, naringenin-7-glucoside, isosakuranin, isosakuranetin, poncirin, astilbin, dihydrokaempferol, and taxifolin), 5 flavones (linarin, scutellarein, cynaroside, luteolin, and narcissin), 4 flavonols (baimaside, tiliroside, astragalin, and spiraeoside), and 9 other flavonoid metabolites. In addition, we analyzed the trends of flavonoid metabolite accumulation with increasing aluminum concentrations. The content of the 24 differential flavonoid metabolites gradually increased with increasing concentrations of aluminum stress, including catechin, epicatechin, naringenin-7-glucoside, astilbin, taxifolin, miquelianin, quercitrin, and quercimeritrin. Nagata et al. [44] determined that most Al binds to catechins, and the release of catechols, catechins, and quercetin in maize has the potential to detoxify aluminum [45]. However, epicatechin is a derivative of catechin, (−)-gallocatechin gallate, and (−)-gallocatechin; moreover, miquelianin, quercitrin, and quercimeritrin are derivatives of quercetin that can be interconverted. Studies have confirmed that epigallocatechin gallate (EGCG) ultimately reduces the mobility and toxicity of aluminum by binding to it, thus forming EGCG-Al complexes in tea [45]. The decrease in (−)-gallocatechin gallate and (−)-gallocatechin under 4 mmol/L Al stress treatment may be caused by the conversion to EGCG and the formation of EGCG-Al complexes, which result in low accumulation of (−)-gallocatechin gallate and (−)-gallocatechin and high accumulation of (−)-catechin gallate, catechin, and epicatechin.

Flavonoids belong to a group of phenolic compounds with a common structure, consisting of two aromatic rings (A and B), and the aromatic rings are bound together by three carbon atoms to form an oxygen-containing heterocycle (Ring C) [46]. There is a growing body of evidence demonstrating that the oxidative stress caused by high levels of metal ions can be removed by phenolic compounds acting as antioxidants, thus protecting plants from damage [26,31]. The most significant increase in antioxidant activity (about 30%) was observed in *C. drupifera* precultured in leaf extracts containing 7.5 and 15 μg/mL of active flavonoids. After preincubation and flavonoid preparation, there was consistently higher antioxidant activity in the treated leaf samples than in the non-stressed controls. Based on the results of the antioxidant activity assay, it can be concluded that flavonoids can act as antioxidants and protect cells from metal ion stress. A prominent feature of phenolic compounds is their complexation with metal ions, such as Al^3+^, and Fu et al. [37] demonstrated that flavonoids have characteristics similar to other phenolic compounds. Our next step is to use ^27^Al nuclear magnetic resonance (NMR) experiments to verify whether Al detoxification in oil tea plants also occurs via the formation of Al complexes, similarly to *C. sinensis*.

## 4. Materials and Methods

### 4.1. Plant Materials and Treatments

In March 2022, two-year-old live seedlings of *C. drupifera* were transplanted into plastic pots (bottom diameter = 18 cm; height = 16 cm; top diameter = 23 cm) for cultivation. After two months of growth, healthy materials with the same growth conditions were selected for aluminum stress treatment. The experimental design consisted of a control (deionized water, CK) and the aluminum treatments (2 and 4 mmol/L of analytically pure AlCl_3_-6H_2_O, GZ2 and GZ4, respectively). AlCl_3_-6H_2_O was dissolved with deionized water, then configured into a solution made up of corresponding concentrations. During the treatment period, *C. drupifera* seedlings were transferred to an artificial climate chamber set to a daytime temperature of 28 °C and a nighttime temperature of 25 °C, adjusted light intensity of 600 µmol m^−2^ s^−1^, and relative humidity of about 75%, and plants were watered with 200 mL of deionized water (CK) or the respective solution every two days (GZ2 and GZ4) under the same conditions. Each treatment contained three biological replicates. After four weeks of aluminum stress treatment, leaves of *C. drupifera* from each treatment were taken for metabolomic analysis of flavonoids.

### 4.2. Sample Preparation and Extraction

*Camellia drupifera* leaves were freeze-dried, ground into powder (35 Hz, 2 min), and stored at −80 °C until use. The sample preparation and extraction procedures were performed as described by Wang et al. [17]. The detection of flavonoid content was conducted by MetWare (http://www.metware.cn/, accessed on 12 March 2022) using the AB Sciex QTRAP 6500 LC-MS/MS platform.

### 4.3. UPLC Conditions

The sample extracts were analyzed using an UPLC-ESI-MS/MS system according to Huang et al. [47]. The analytical conditions were set according to those reported by Zhang et al. [48]. In brief, for the ultra-performance liquid chromatograph, an Agilent SB-C18 column, Santa Clara, CA, USA (1.8 μm, 2.1 × 100 mm) was used. Sample measurements were performed using a gradient program in which the starting conditions were 95% A (pure water with 0.1% formic acid) and 5% B (acetonitrile with 0.05% formic acid). The gradient elution program was set as described by Wang et al. [17].

### 4.4. ESI-MS/MS Conditions

The ESI source operation parameters were set according to those reported by Wang et al. [17]. In brief, linear ion trap (LIT) and triple quadrupole (QQQ) scans were acquired on a triple quadrupole-linear ion trap mass spectrometer (QTRAP), QTRAP^®^ 6500+ LC-MS/MS System equipped with an ESI Turbo Ion-Spray interface, operating in positive and negative ion mode and controlled by Analyst 1.6.3 software (Sciex). Flavonoid data were collected and identified by scheduled multiple reaction monitoring (MRM) according to Wang et al. [17]. MultiQuant 3.0.3 software (Sciex, Toronto, IL, Canada) was used to quantify all the metabolites. The parameters of mass spectrometry, including the depolymerization potential (DP) and collision energy (CE) of individual MRM transitions, were applied with further optimization of the DP and CE [49,50]. A specific set of MRM transitions was monitored during each period based on the eluted metabolites [49,50]. The MWDB database, constructed using standards, was used for qualitative analysis of the data detected by mass spectrometry.

### 4.5. Multivariate Statistical Analysis

PCA analysis is an innovative multivariate statistical method based on the premise that only a few principal components are sufficient to reveal the internal differences between multiple variables [36]. Unsupervised principal component analysis was performed across all samples using the log_2_-normalized metabolite expression levels. Unit variance scaling was performed with the data before the unsupervised PCA. Orthogonal partial least squares discriminant analysis (OPLS-DA) was used to predict the stability and reliability of the models. Metabolites from nine samples were used for hierarchical clustering analysis (HCA) and compared via Venn diagrams. The HCA results of *C. drupifera* samples and flavonoid metabolites were presented as heatmaps with dendrograms, while the Pearson correlation coefficients (PCC) between samples were calculated by the cor function in R (www.rproject.org/, accessed on 8 May 2022) and presented only as heatmaps. Both HCA and PCC were carried out by R (R version 3.5.0) package pheatmap. For HCA, normalized signal intensities of metabolites (unit variance scaling) were visualized as a color spectrum. K-means cluster analyses were performed using the ‘apcluster’ software package implemented in the free statistical application R.

Flavonoid metabolites that significantly changed between groups were determined on the basis of variable importance in projection (VIP) ≥1 and absolute log_2_FC (fold change) ≥1.0. VIP values were extracted from the OPLS-DA results, which were also determined through score plots and permutation plots, and then generated using the R package MetaboAnalystR. The differential flavonoid metabolites of *C. drupifera* under aluminum stress were annotated using the Kyoto Encyclopedia of Genes and Genomes (KEGG) database. Pathways for which significantly regulated metabolites were mapped were then fed into metabolite set enrichment analysis; their significance was determined according to a hypergeometric test’s *p*-values.

### 4.6. Antioxidative Activity

The scavenging activity of flavonoids against diphenylpicrylhydrazyl (DPPH) was determined according to the method proposed by Martínez-Villaluenga et al. [51], with minor modifications. In brief, the leaves were extracted with 80% aqueous methanol (1 g/10 mL), shaking at room temperature for 2 h. DPPH was weighed and prepared in 0.04 mg/mL of DPPH solution with anhydrous ethanol. The mixture was placed at room temperature for 30 min and centrifuged at 13,000× *g* for 10 min, then the supernatant was removed. The scavenging activity of the test compound was calculated as the degree of the decrease in DPPH absorbance at 517 nm, and was expressed as a percentage of the absorbance of a control DPPH solution without the test compound [26]. The percentages of inhibition of the DPPH radical, as a function of the effect of the extracted fractions, were calculated according to the methods of Izbiańska et al. [26] and Molynex [52].

### 4.7. Quantitative Real-Time PCR (qRT-PCR)

qRT-PCR was used to detect differences in the relative expression of the genes encoding the key enzymes involved in flavonoid synthesis (*IFS*, *F3H*, *DFR*, *FLS*, *CHS1,* and *PAL*) in *C. drupifera* under different concentrations of Al. Total RNA was extracted by grinding 5~200 mg of leaf tissue to a powder in liquid nitrogen, and the total RNA concentration was determined according to the protocol of Singh et al. [53] with minor modifications. cDNA was synthesized by reverse transcription of total RNA, according to the procedures of Singh et al. [53], and stored at −20 °C. The expression levels of the *IFS*, *F3H*, *DFR*, *FLS*, *CHS*1, and *PAL* genes were measured via qRT-PCR, with the β-tubulin gene used as an internal reference. Each reaction was repeated three times with three biological replicates, and the results were analyzed using the 2^−ΔΔCt^ method. The reaction system was based on that in the study of Singh et al. [53], and the sequences of the primers, which were synthesized by Bioengineering (Shanghai, China) Co., are shown in Table 2.

## 5. Conclusions

In conclusion, this study focused on Al resistance mechanisms involving flavonoid metabolites in oil tea plants. The results demonstrate that different concentrations of Al exert a significant influence on the metabolites of *C. drupifera*; 40 flavonoid metabolites were found to be significantly upregulated and 9 downregulated under Al stress relative to the control group, and the content of the 24 differential flavonoid metabolites was gradually upregulated with increasing concentrations of Al stress, including of catechin, epicatechin, naringenin-7-glucoside, astilbin, taxifolin, miquelianin, quercitrin, and quercimeritrin. Moreover, the most significant increase in antioxidant activity (about 30%) was observed in *C. drupifera* precultured in leaf extracts containing 7.5 and 15 μg/mL of active flavonoids. The qRT-PCR results showed that the expression levels of key genes involved in the synthesis of flavonoids were consistent with the accumulation trends of flavonoids among the different Al treatments. The mechanisms of Al tolerance and the accumulation of Al in oil tea plants are complex and require additional clarification. For instance, whether the mechanism of flavonoids involves forming complexes with Al and specific genes and proteins related to the synthesis of flavonoid compounds for the growth of oil tea plants should be addressed in further research.

## Figures and Tables

**Table 1:** The 49 differential flavonoid metabolites in GZ2 vs. CK and GZ4 vs. CK.

Index	Compounds	Molecular Weight	Formula	VIP	*p*-Value	Trends	
						GZ2/CK	GZ4/CK
Flavonoid_22	Neohesperidin dihydrochalcone	612.21	C_28_H_36_O_15_	1.20	0.0018	Up	Up
Flavonoid_80	(−)-Gallocatechin gallate	458.08	C_22_H_18_O_11_	1.17	0.0236	Down	Down
Flavonoid_78	Eriocitrin	596.17	C_27_H_32_O_15_	1.22	0.0005	Up	Up
Flavonoid_101	Hesperidin	610.19	C_28_H_34_O_15_	1.21	0.0262	Down	Down
Flavonoid_48	Narirutin	580.18	C_27_H_32_O_14_	1.22	0.0015	Up	Up
Flavonoid_176	Naringenin-7-glucoside	434.12	C_21_H_22_O_10_	1.19	0.0003	Up	Up
Flavonoid_141	Isosakuranetin	448.14	C_22_H_24_O_10_	1.11	0.0078	Up	Up
Flavonoid_01	Astilbin	450.12	C_21_H_22_O_11_	1.20	0.0064	Up	Up
Flavonoid_139	Dihydrokaempferol	288.06	C_15_H_12_O_6_	1.22	0.0021	Up	Up
Flavonoid_56	Taxifolin	304.06	C_15_H_12_O_7_	1.21	0.0000	Up	Up
Flavonoid_64	Scutellarein	286.05	C_15_H_10_O_6_	1.22	0.0031	Up	Up
Flavonoid_90	Cynaroside	448.10	C_21_H_20_O_11_	1.21	0.0140	Up	Up
Flavonoid_67	Fisetin	286.05	C_15_H_10_O_6_	1.04	0.0205	Up	Up
Flavonoid_194	Quercimeritrin	464.10	C_21_H_20_O_12_	1.18	0.0057	Up	Up
Flavonoid_02	Miquelianin	478.07	C_21_H_18_O_13_	1.21	0.0020	Down	Down
Flavonoid_119	Astragalin	448.10	C_21_H_20_O_11_	1.21	0.0042	Up	Up
Flavonoid_180	Spiraeoside	464.10	C_21_H_20_O_12_	1.21	0.0031	Up	Up
Flavonoid_152	Ononin	430.13	C_22_H_22_O_9_	1.20	0.0034	Up	Up
Flavonoid_114	6′-O-Acetylglycitin	488.13	C_24_H_24_O_11_	1.21	0.1004	Up	Up
Flavonoid_102	Theaflavin	564.13	C_29_H_24_O_12_	1.22	0.0013	Down	-
Flavonoid_150	(-)-Catechin gallate	442.09	C_22_H_18_O_10_	1.13	0.0360	Up	-
Flavonoid_108	Pinocembrin	256.07	C_15_H_12_O_4_	1.12	0.0866	Up	-
Flavonoid_158	Isosakuranetin	286.08	C_16_H_14_O_5_	1.21	0.0044	Up	-
Flavonoid_179	Poncirin	594.19	C_28_H_34_O_14_	1.21	0.0040	Up	-
Flavonoid_204	Linarin	592.18	C_28_H_32_O_14_	1.22	0.0008	Up	-
Flavonoid_50	Luteolin	286.05	C_15_H_10_O_6_	1.19	0.0089	Up	-
Flavonoid_118	Narcissin	624.17	C_28_H_32_O_16_	1.21	0.0014	Down	-
Flavonoid_86	Avicularin	434.08	C_20_H_18_O_11_	1.20	0.0007	Up	-
Flavonoid_138	Baimaside	626.15	C_27_H_30_O_17_	1.21	0.0009	Up	-
Flavonoid_137	Tiliroside	594.14	C_30_H_26_O_13_	1.21	0.0001	Up	-
Flavonoid_65	Phlorizin	436.14	C_21_H_24_O_10_	1.05	0.0736	-	Down
Flavonoid_201	Trilobatin	436.14	C_21_H_24_O_10_	1.15	0.0123	-	Up
Flavonoid_177	Phloretin	274.08	C_15_H_14_O_5_	1.15	0.0027	-	Down
Flavonoid_191	Naringenin chalcone	272.07	C_15_H_12_O_5_	1.16	0.0000	-	Up
Flavonoid_55	(−)-Catechin	290.08	C_15_H_14_O_6_	1.15	0.0001	-	Up
Flavonoid_147	(−)-Epicatechin	290.08	C_15_H14O6	1.16	0.0016	-	Up
Flavonoid_195	(−)-Gallocatechin	306.07	C_15_H_14_O_7_	1.03	0.1439	-	Down
Flavonoid_126	Eriodictyol	288.06	C_15_H_12_O_6_	1.16	0.0021	-	Up
Flavonoid_124	Silychristin	482.12	C_25_H_22_O_10_	1.16	0.0000	-	Up
Flavonoid_40	Dihydromyricetin	320.05	C_15_H_12_O_8_	1.01	0.0187	-	Up
Flavonoid_117	Engeletin	434.12	C_21_H_22_O_10_	1.15	0.0005	-	Up
Flavonoid_154	Vitexin	432.11	C_21_H_20_O_10_	1.13	0.0236	-	Up
Flavonoid_115	Quercitrin	448.10	C_21_H_20_O_11_	1.16	0.0001	-	Up
Flavonoid_106	Typhaneoside	770.23	C_34_H_42_O_20_	1.08	0.0312	-	Up
Flavonoid_57	Rutin	610.15	C_27_H_30_O_16_	1.16	0.0000	-	Up
Flavonoid_197	Isorhamnetin 3-O-glucoside	478.11	C_22_H_22_O_12_	1.16	0.0000	-	Up
Flavonoid_43	Kaempferol	286.05	C_15_H_10_O_6_	1.16	0.0464	-	Down
Flavonoid_175	Afzelin	432.11	C_21_H_20_O_10_	1.16	0.0000	-	Up
Flavonoid_73	Genistin	432.11	C_21_H_20_O_10_	1.02	0.0151	-	Up

Note: Fold changes of ≥2 or ≤0.5 and VIP ≥ 1 were considered to denote significant differences and were used as standards for screening the metabolites. ‘-’ indicates no significant difference.

**Table 2:** Primers for qRT-PCR.

Genes	Upstream Primers (5′→3′)	Downstream Primers (5′→3′)	Reference
*IFS*	ACAACGGCGGAACATACG	ACACTGCTTGCCACTCACC	[54]
*F3H*	AAGAAGTGGAGCAAGGGAAAG	CGGAGACATTGGTGGAGAAA	[54]
*DFR*	ATGTTGCTACACCGTGGTTAC	AAATCGAAAGATCCCTCCTC	[54]
*FLS*	GCACATGATGCAACGCTACT	GGAACCTCTAATTGGGTCCCTC	[27]
*CHS1*	GCCAAGAGTGGTGTGGATAGG	TGCCCTTTGAGCATTGCGAA	[27]
*PAL*	GGAGGATCAACTCCACATAGC	AGTCACTGCTGGCCTTAACTC	[27]
β-Tubulin	ATGTTCAGGCGCAAGGCTT	TCTGCAACCGGGTCATTCAT	[54]

## Data Availability

Not applicable.

## Supplementary Materials

The following supporting information can be downloaded at https://www.mdpi.com/article/10.3390/plants12071432/s1. Table S1: The 78 metabolites that were identified in positive and negative ionization modes. Table S2: The flavonoid metabolites that were identified as significantly changed metabolites.

## References

[1] Zhuang R.L. (2008). Chinese Camellia.

[2] Li Y., Liao B., Wang Y., Luo H., Wang S., Li C., Song W., Zhang K., Yang B., Lu S. (2022). Transcriptome and metabolome analyses provide insights into the relevance of pericarp thickness variations in *Camellia drupifera* and *Camellia oleifera*. Front. Plant Sci..

[3] Qin S., Rong J., Zhang W., Chen J. (2018). Cultivation history of *Camellia oleifera* and genetic resources in the Yangtze River Basin. Biodivers. Sci..

[4] Yao X.H. (2016). Oil-Tea Camellia Cultivars in China.

[5] Delhaize E., Ryan P.R. (1995). Aluminum Toxicity and Tolerance in Plants. Plant Physiol..

[6] Ma J.F., Chen Z.C., Shen R.F. (2014). Molecular mechanisms of Al tolerance in gramineous plants. Plant Soil.

[7] Kouki R., Ayachi R., Ferreira R., Sleimi N. (2021). Behavior of *Cucumis sativus* L. in presence of aluminum stress: Germination, plant growth, and antioxidant enzymes. Food Sci. Nutr..

[8] Ma L., Yang S. (2022). Growth and physiological response of *Kandelia obovata* and *Bruguiera sexangula* seedlings to aluminum stress. Environ. Sci. Pollut. Res..

[9] Zhang H.L., Arnall B., Hiner G. (2017). Problem Soils. Oklahoma Soil Fertility Handbook.

[10] Kochian L.V., Hoekenga O.A., Piñeros M.A. (2004). How do crop plants tolerate acid soils? Mechanisms of aluminum tolerance and phosphorous efficiency. Annu. Rev. Plant Biol..

[11] Huang L., Yuan J., Wang H., Tan X., Niu G. (2017). Aluminum Stress Affects Growth and Physiological Characteristics in Oil Tea. Hortscience.

[12] Zhou J., Ai Z.Z., Wang H.L., Niu G.H. (2019). Phosphorus Alleviates Aluminum Toxicity in *Camellia oleifera* Seedlings. Int. J. Agric. Biol..

[13] Qu X., Zhou J., Masabni J., Yuan J. (2020). Phosphorus relieves aluminum toxicity in oil tea seedlings by regulating the metabolic profiling in the roots. Plant Physiol. Biochem..

[14] Brunetti C., George R.M., Tattini M., Field K., Davey M.P. (2013). Metabolomics in plant environmental physiology. J. Exp. Bot..

[15] Ferdinando M.D., Brunetti C., Fini A., Tattini M. (2012). Flavonoids as Antioxidants in Plants under Abiotic Stresses.

[16] Qu X., Hu S., Li T., Zhang J., Wang B., Liu C. (2022). Metabolomics Analysis Reveals the Differences Between *Bupleurum chinense* DC. and *Bupleurum scorzonerifolium* Willd. Front. Plant Sci..

[17] Wang Y., Cheng J., Jiang W., Chen S. (2022). Metabolomics study of flavonoids in *Coreopsis tinctoria* of different origins by UPLC-MS/MS. PeerJ.

[18] Ferreyra M.L.F., Rius S.P., Casati P. (2012). Flavonoids: Biosynthesis, biological functions, and biotechnological applications. Front. Plant Sci..

[19] Pan J., Vicente A.R., Martínez G.A., Chaves A.R., Civello P.M. (2004). Combined use of UV-C irradiation and heat treatment to improve postharvest life of strawberry fruit. J. Sci. Food Agric..

[20] Chutipaijit S., Cha-Um S., Sompornpailin K. (2009). Differential accumulations of proline and flavonoids in indica rice varieties against salinity. Pak. J. Bot..

[21] Li B., Fan R., Sun G., Sun T., Fan Y., Bai S., Guo S., Huang S., Liu J., Zhang H. (2021). Flavonoids improve drought tolerance of maize seedlings by regulating the homeostasis of reactive oxygen species. Plant Soil.

[22] Rivero R.M., Ruiz J.M., García P.C., López-Lefebre L.R., Sánchez E., Romero L. (2001). Resistance to cold and heat stress: Accumulation of phenolic compounds in tomato and watermelon plants. Plant Sci..

[23] He J., Yao L., Pecoraro L., Liu C., Wang J., Huang L., Gao W. (2022). Cold stress regulates accumulation of flavonoids and terpenoids in plants by phytohormone, transcription process, functional enzyme, and epigenetics. Crit. Rev. Biotechnol..

[24] Kohli S.K., Handa N., Sharma A., Gautam V., Arora S., Bhardwaj R., Wijaya L., Alyemeni M.N., Ahmad P. (2018). Interaction of 24-epibrassinolide and salicylic acid regulates pigment contents, antioxidative defense responses, and gene expression in *Brassica juncea* L. seedlings under Pb stress. Environ. Sci. Pollut. Res..

[25] Kaur R., Yadav P., Sharma A., Thukral A.K., Kumar V., Kohli S.K., Bhardwaj R. (2017). Castasterone and citric acid treatment restores photosynthetic attributes in *Brassica juncea* L. under Cd(II) toxicity. Ecotoxicol. Environ. Saf..

[26] Izbiańska-Jankowska K., Arasimowicz-Jelonek M., Deckert J. (2014). Phenylpropanoid pathway metabolites promote tolerance response of lupine roots to lead stress. Ecotoxicol. Environ. Saf..

[27] Su L., Lv A., Wen W., Fan N., Li J., Gao L., Zhou P., An Y. (2022). MsMYB741 is involved in alfalfa resistance to aluminum stress by regulating flavonoid biosynthesis. Plant J..

[28] Babu T.S., Akhtar T.A., Lampi M.A., Tripuranthakam S., Dixon D.G., Greenberg B.M. (2003). Similar Stress Responses are Elicited by Copper and Ultraviolet Radiation in the Aquatic Plant Lemna gibba: Implication of Reactive Oxygen Species as Common Signals. Plant Cell Physiol..

[29] Sobkowiak R., Deckert J. (2006). Proteins induced by cadmium in soybean cells. J. Plant Physiol..

[30] Pawlak-Sprada S., Arasimowicz-Jelonek M., Podgórska M., Deckert J. (2011). Activation of phenylopropanoid pathway in legume plants exposed to heavy metals. Part I. Effects of cadmium and lead on phenylalanine ammonia-lyase gene expression, enzyme activity and lignin content. Acta Biochim. Pol..

[31] Pawlak-Sprada S., Stobiecki M., Deckert J. (2011). Activation of phenylpropanoid pathway in legume plants exposed to heavy metals. Part II. Profiling of isoflavonoids and their glycoconjugates induced in roots of lupine (*Lupinus luteus*) seedlings treated with cadmium and lead. Acta Biochim. Pol..

[32] Xu Q., Wang Y., Ding Z., Fan K., Ma D., Zhang Y., Yin Q. (2017). Aluminum induced physiological and proteomic responses in tea (*Camellia sinensis*) roots and leaves. Plant Physiol. Biochem..

[33] Li B.Z., Fan R.N., Fan Y.T., Liu R.N., Zhang H., Chen T.T., Liu J., Li H., Zhao X., Song C.P. (2022). The flavonoid biosynthesis regulator PFG3 confers drought stress tolerance in plants by promoting flavonoid accumulation. Environ. Exp. Bot..

[34] Tahara K., Hashida K., Otsuka Y., Ohara S., Kojima K., Shinohara K. (2013). Identification of a Hydrolyzable Tannin, Oenothein B, as an Aluminum-Detoxifying Ligand in a Highly Aluminum-Resistant Tree, *Eucalyptus camaldulensis*. Plant Physiol..

[35] Osawa H., Endo I., Hara Y., Matsushima Y., Tange T. (2010). Transient Proliferation of Proanthocyanidin-Accumulating Cells on the Epidermal Apex Contributes to Highly Aluminum-Resistant Root Elongation in Camphor Tree. Plant Physiol..

[36] Li J., Hossain S., Ma H., Yang Q., Gong X., Yang P., Feng B. (2019). Comparative Metabolomics Reveals Differences in Flavonoid Metabolites among Different Coloured Buckwheat Flowers. J. Food Compos. Anal..

[37] Fu Z., Jiang X., Li W.-W., Shi Y., Lai S., Zhuang J., Yao S., Liu Y., Hu J., Gao L. (2020). Proanthocyanidin-Aluminum Complexes Improve Aluminum Resistance and Detoxification of *Camellia sinensis*. J. Agric. Food Chem..

[38] Xu Q., Wang Y., Ding Z., Song L., Li Y., Ma D., Wang Y., Shen J., Jia S., Sun H. (2016). Aluminum Induced Metabolic Responses in Two Tea Cultivars. Plant Physiol. Biochem..

[39] Ryan P.R., Tyerman S., Sasaki T., Furuichi T., Yamamoto Y., Zhang W.H., Delhaize E. (2010). The Identification of Aluminium-Resistance Genes Provides Opportunities for Enhancing Crop Production on Acid Soils. J. Exp. Bot..

[40] Xu L.M., Chan L.I.U., Cui B.M., Ning W.A.N.G., Zhuo Z.H.A.O., Zhou L.N., Huang K.F., Ding J.Z., Du H.M., Jiang W. (2018). Transcriptomic Responses to Aluminum (Al) Atress in Maize. J. Integr. Agric..

[41] Pourcel L., Routaboul J.M., Cheynier V., Lepiniec L., Debeaujon I. (2007). Flavonoid Oxidation in Plants: From Biochemical Properties to Physiological Functions. Trends Plant Sci..

[42] Brunetti C., Fini A., Sebastiani F., Gori A., Tattini M. (2018). Modulation of Phytohormone Signaling: A Primary Function of Flavonoids in Plant-Environment Interactions. Front. Plant Sci..

[43] Ofei-Manu P., Wagatsuma T., Ishikawa S., Tawaraya K. (2001). The Plasma Membrane Strength of the Root-tip Cells and Root Phenolic Compounds are Correiated with Al Tolerance in Several Common Woody Plants. Soil Sci. Plant Nutr..

[44] Nagata T., Hayatsu M., Kosuge N. (1992). Identification of Aluminium Forms in Tea Leaves by ^27^Al NMR. Phytochemistry.

[45] Kidd P.S., Llugany M., Poschenrieder C., Gunsé B., Barceló J. (2001). The Role of Root Exudates in Aluminium Resistance and Siliconinduced Amelioration of Aluminium Toxicity in Three Varieties of Maize (*Zea mays* L.). J. Exp. Bot..

[46] Mutha R.E., Tatiya A.U., Surana S.J. (2021). Flavonoids as Natural Phenolic Compounds and their Role in Therapeutics: An Overview. Future J. Pharm. Sci..

[47] Huang D., Ming R., Yao S., Li L., Huang R., Tan Y. (2021). Identification of Anthocyanins in the Fruits of *Kadsura coccinea* using UPLC-MS/MS-based Metabolomics. Biochem. Syst. Ecol..

[48] Zhang Y., Gao J., Qie Q., Yang Y., Hou S., Wang X., Li X., Han Y. (2021). Comparative Analysis of Flavonoid Metabolites in Foxtail Millet (*Setaria italica*) with Different Eating Quality. Life.

[49] Li L., Teng J., Zhu Y., Xie F., Hou J., Ling Y., Zhu H. (2021). Metabolomics Study of Flavonoids of *Taxilluschinensis* on Different Hosts Using UPLC-ESI-MS/MS. Molecules.

[50] Liang S., Wen Z., Tang T., Liu Y., Dang F., Xie T., Wu H. (2021). Study on Flavonoid and Bioactivity Features of the Pericarp of *Citri Reticulatae* ‘chachi’ during Storage. Arab. J. Chem..

[51] Martínez-Villaluenga C., Zieliński H., Frias J., Piskula M.K., Kozłowska H., Vidal-Valverde C. (2009). Antioxidant Capacity and Polyphenolic Content of High-protein Lupin Products. Food Chem..

[52] Molynex P. (2004). The use of the stable free radical diphenylpicrylhydrazyl (DPPH) for estimating antioxidant activity. J. Sci. Technol..

[53] Singh P., Singh R.K., Song Q.-Q., Li H.-B., Guo D.-J., Malviya M.K., Verma K.K., Song X.-P., Lakshmanan P., Yang L.T. (2021). Comparative Analysis of Protein and Differential Responses of Defense-related Gene and Enzyme Activity Reveals the Long-term Molecular Responses of Sugarcane Inoculated with *Sporisorium scitamineum*. J. Plant Interact..

[54] Wang Y., Jiang W., Cheng J.S., He X.G., Chu G.H. (2022). Differential Analysis of Flavonoid Metabolites and Key Enzyme Gene Ex-pressions in *Coreopsis tinctoria* Collected from Plateau and Plain Areas. J. Food Saf. Food Qual..

